# Supplementary material for: The genome sequence of the muscid fly Eudasyphora cyanicolor (Zetterstedt, 1845)
Source: Wellcome Open Res. 2025 Feb 10;10:56. [Version 1] doi: 10.12688/wellcomeopenres.23675.1 (PMC11868753; doi:10.12688/wellcomeopenres.23675.1)
Supplement: Peer review history

# Reviewer report: Reviewer response for version 1

**DOI:** 10.21956/wellcomeopenres.26118.r118406

**Published:** 2025-02-27

**Authors:** Rakesh K Mishra

**Affiliations:** 1 Tata Institute for Genetics and Society, Bengaluru, Karnataka, India

Authors in this report present genome sequencing and assembly of muscid fly. They also use NGS based approach to assemble the sequence data in five molecules (chromosomes). The description of the process and approach is described in sufficient details. This will be a very useful information in the fast growing field of genome biology of insects for understanding basic biology and human health perspective as the information is of potential use in genome based technologies of vector control.

Are sufficient details of methods and materials provided to allow replication by others?

Yes

Is the rationale for creating the dataset(s) clearly described?

Yes

Are the datasets clearly presented in a useable and accessible format?

Yes

Are the protocols appropriate and is the work technically sound?

Yes

Reviewer Expertise:

Genome organisation, comparative genomics, genetics and epigenetic regulation of genes

I confirm that I have read this submission and believe that I have an appropriate level of expertise to confirm that it is of an acceptable scientific standard.

# Reviewer report: Reviewer response for version 1

**DOI:** 10.21956/wellcomeopenres.26118.r119418

**Published:** 2025-02-26

**Authors:** Jason Charamis

**Affiliations:** 1 Foundation for Research and Technology - Hellas, Irákleion, Greece

This article describes the genome sequencing of the housefly *Eudasyphora cyanicolor*. The study produced a highly contiguous and highly accurate genome assembly of all main chromosomes, as demonstrated by the BUSCO analysis and the k-mer based QV metric, except for the sex chromosomes. This is a common challenge in dipteran genome assemblies, due to the frequent evolutionary turnover of sex chromosomes in this order.

 The manuscript is mostly ready for indexing. My only request is to better describe the relevance of sequencing of this species for future ecological studies. For example, because of its synanthropic behavior, this species has been probably exposed to pesticides for several decades. In this context, genome sequencing would be useful for studying insecticide resistance in non-target insects. I would like to see the Introduction section expanded to better establish the relevance of this genome assembly.

Are sufficient details of methods and materials provided to allow replication by others?

Yes

Is the rationale for creating the dataset(s) clearly described?

Yes

Are the datasets clearly presented in a useable and accessible format?

Yes

Are the protocols appropriate and is the work technically sound?

Yes

Reviewer Expertise:

Arthropod Comparative Genomics

I confirm that I have read this submission and believe that I have an appropriate level of expertise to confirm that it is of an acceptable scientific standard.
